# Identification of *Cr*DCL1-mediated microRNA biogenesis in green alga *Chlamydomonas reinhardtii*

**DOI:** 10.3389/fmicb.2025.1487584

**Published:** 2025-02-27

**Authors:** Ting Sun, Ming Tao, Qinglang Di, Zhangli Hu, Hui Li, Sulin Lou

**Affiliations:** ^1^Guangdong Key Laboratory of Plant Epigenetics, Guangdong Engineering Research Center for Marine Algal Biotechnology, Longhua Innovation Institute for Biotechnology, College of Life Sciences and Oceanography, Shenzhen University, Shenzhen, China; ^2^Medical School, Shenzhen University, Shenzhen, China; ^3^The Affiliated International School of Shenzhen University, Shenzhen, China

**Keywords:** *Cr*DCL1, microRNA, biogenesis, small RNA-sequencing, *Chlamydomonas reinhardtii*

## Abstract

In eukaryotes, microRNAs (miRNAs) are generated by Dicer/Dicer-Like (DCL)-mediated cleavage. Previous studies identified three DCL genes (*Cr*DCL1-3) in *Chlamydomonas reinhardtii* and indicated that *Cr*DCL3 mediated the production of most miRNAs, while *Cr*DCL1 protein was mainly involved in siRNA biogenesis. The role of *Cr*DCL1 in miRNA biogenesis remains unclear. This study constructed a phylogenetic tree, performed structural analyses of Dicer/DCL proteins from multiple species and screened and verified *dcl1* and *dcl3* mutant strains. Using CC-5325 and *dcl3* mutant as control groups, we performed sRNA-sequencing, RT-qPCR, and Northern blot verification experiments on *dcl1* mutant to explore the involvement of *Cr*DCL1 in miRNA biogenesis in *C. reinhardtii*. The results demonstrated that *Cr*DCL1 was involved in the production of 22 miRNAs, including cre-miR910, novel-miR01, novel-miR03, novel-miR04, novel-miR05, and novel-miR06, whose production was depended not only on *Cr*DCL1 but also on *Cr*DCL3. The present findings highlight the production of some *C. reinhardtii* miRNAs that may be involved in multiple *Cr*DCL proteins, which is different from animals and plants. The results of this study will enrich the knowledge of miRNA biogenesis in eukaryotes.

## 1 Introduction

RNA-mediated silencing in eukaryotes involves highly conserved and specific gene expression regulatory mechanisms. Small RNAs (sRNAs) are incorporated into Piwi/Argonaute (AGO) proteins to form RNA-induced silencing complexes (RISC), which negatively regulate the expression of their target genes through complementary base pairing (Baulcombe, 2004; Meister, 2013). RNA-mediated silencing plays important roles in developmental regulation, genome stability maintenance in response to stress and viral infection (Baulcombe, 2004; Matranga and Zamore, 2007; Casas-Mollano et al., 2008). There are three major classes of sRNAs mediating RNA silencing in eukaryotes, including small interfering RNAs (siRNAs), microRNAs (miRNAs), and Piwi-interacting RNAs (piRNAs) (Matranga and Zamore, 2007; Ghildiyal and Zamore, 2009). Cleavage by the RNase III family enzyme Dicer or Dicer-like proteins produces siRNAs and miRNAs. The production of piRNAs is unclear, but it is independent from Dicer proteins (Iwasaki et al., 2015).

miRNAs are 18–24 nucleotide (nt) non-coding sRNAs that induce target mRNA cleavage or repress target mRNA translation at the post-transcriptional level. Following the initial discovery of miRNAs in *Caenorhabditis elegans* in 1993 (Lee et al., 1993), miRNAs were identified in other multicellular eukaryotes, such as *Arabidopsis*, *Drosophila* and humans. Subsequently, miRNAs were also identified in unicellular organisms later (Lou et al., 2018). In animals, miRNA biogenesis begins with the production of primary transcripts (pri-miRNAs) by RNA polymerase II. These pri-miRNAs are folded into imperfect stem-loop structures and processed to generate precursor-miRNAs (pre-miRNAs) in the nucleus by an RNase III enzyme called Drosha (Han et al., 2004). The pre-miRNAs are transported to the cytoplasm by Exportin 5, followed by cleavage of the loop by the RNase III enzyme Dicer to release the 18–24 nt miRNA/miRNA* duplex (Yi et al., 2003; Lund et al., 2004; Bartel, 2009). By contrast, the generation of plant miRNA/miRNA* duplexes occurs entirely within the nucleus, typically with two cleavage steps executed by an individual Dicer-like (DCL) RNase III enzyme. Plant miRNA/miRNA* duplexes are then transported to the cytoplasm and stabilized through HEN1 methylation (Kurihara and Watanabe, 2004; Kurihara et al., 2006; Eamens et al., 2009). In *Arabidopsis thaliana*, four Dicer homologs (*At*DCL1-4) are discovered. *At*DCL1 is mainly involved in miRNA biogenesis and *At*DCL2-4 are involved in the biogenesis of various types of siRNAs. Notably, a few miRNAs such as miR822, miR839 and miR859 differ from other miRNAs in terms of their biogenesis: they are produced independently from *At*DCL1 and rely on the cleavage by *At*DCL4 (Rajagopalan et al., 2006; Ben et al., 2009; Tsuzuki et al., 2014; Yu et al., 2017).

miRNAs have been firstly reported in the single-cell alga *Chlamydomonas reinhardtii* in 2007 (Zhao et al., 2007; Molnár et al., 2007). Among the three *Cr*DCLs (*Cr*DCL1-3) in *C. reinhardtii*, *Cr*DCL3 is involved in the production of most miRNAs with the assistance of *Cr*DUS16 (Casas-Mollano et al., 2008; Merchant et al., 2007; Valli et al., 2016; Yamasaki et al., 2016). Casas-Mollano et al. demonstrated that CrDCL1 mediates siRNA accumulation and post-transcriptional silencing of the TOC1 retrotransposon (Casas-Mollano et al., 2008). The function of the *Cr*DCL2 protein remains unknown. It is unclear whether the cleavage of pre-miRNAs occurs in the nucleus or cytoplasm, and whether the *Cr*DCL1/*Cr*DCL2 proteins play a role in the production of individual miRNAs similar to *At*DCL4. This study aimed to verified whether *Cr*DCL1 is also involved in miRNA processing, through sRNA high-throughput sequencing, using the *dcl1* mutant. In comparison with the wild type CC-5325, differentially expressed miRNAs were identified and validated by real-time quantitative PCR (RT-qPCR) and Northern blot. By further evaluating the amount of differentially expressed miRNA in the *dcl3* mutant, the results will figure out the role of *Cr*DCL1 in miRNA biogenesis.

## 2 Materials and methods

### 2.1 Cultivation and screening of *C. reinhardtii* strains

The wild type CC-5325, the *dcl1* mutant strains (ID: LMJ.RY0402.124662; LMJ.RY0402.198146), and the *dcl3* mutant strains (ID: LMJ.RY0402.253048; LMJ.RY0402.080558) were obtained from the Chlamydomonas Library Project (CLiP).^1^ These mutants were generated by random insertion of CIB1, a 2,223 bp double-stranded DNA transformation cassette, into the strain CC-5325 by electroporation (Zhang et al., 2014). The transformation cassette is composed of two random sequences, a *PSAD* promoter, a *HSP70-RBCS2* promoter, a *RBCS2* intron, the *AphVIII* gene (conferring paromomycin resistance), a *PSAD* terminator and the *RPL12* terminator in the opposite direction to block transcription of the target gene.^2^ The *dcl1* and *dcl3* mutants exclusively harbored mutations in the *CrDCL1* and *CrDCL3* gene, respectively, without affecting other genes. Consequently, these mutants were chosen to investigate the functions of the *CrDCL1* and *CrDCL3* genes. *C. reinhardtii* cells were grown in Tris-acetate-phosphate (TAP) medium at 22°C under continuous illumination (100 μE m^−2^·s^−1^) and were aerated daily by shaking the bottles twice. For *dcl1* and *dcl3* mutants, the TAP medium was supplemented with 10 μg ml^−1^ paromomycin (Li et al., 2016; Zhang et al., 2014).

To confirm the mutants, colony PCR was performed. The genomic DNA was extracted from single colony obtained by streak cultivation, using Ultra DNA Isolation Kit (BEI-BEI BIOTECH). Primers used to verify the presence of 2,223 bp transformation cassette in mutants were listed in Supplementary Table S1.

### 2.2 Bioinformatic analysis of DCL proteins

The amino acid sequences of three *C. reinhardtii* DCL proteins (*Cr*DCL1-3, gene IDs: *Cre02.g141000*, *Cre16.g684715*, *Cre07.g345900*), four *Arabidopsis thaliana* DCL proteins (*At*DCL1-4, gene IDs: *AT1G01040.2*, *AT3G03300.1*, *AT3G43920.2*, *AT5G20320.1*), and two *Oryza sativa* putative DCL proteins (*Os*DCL2-3, gene IDs: *LOC_Os09g14610.1*, *LOC_Os01g68120.1*) were obtained from Phytozome (v12.1).^3^ The amino acid sequences of Dicer proteins from *Mus musculus* (*Mm*) (GenBank accession: NP_001398758.1), *Homo sapiens* (*Hs*) (GenBank accession: NP_001182502.1), *Drosophila melanogaster* (*Dm*) (GenBank accessions: AAF56056.1, AAF57830.2), *Rhodotorula toruloides* (*Rt*) (GenBank accession: XP_016275102.1), and *Schizosaccharomyces pombe* (*Sp*) (GenBank accession: NP_588215.2), as well as the amino acid sequences of Drosha protein from *Cricetulus griseus* (*Cg*) (GenBank accession: XP_035296509.1), *Caenorhabditis elegans* (*Ce*) (GenBank accession: O01326.2) and *D. melanogaster* (GenBank accession: AAF59169.1) were obtained from NCBI database.^4^ All sequences were aligned using ClustalW in MEGA X software and subsequently trimmed using trimAl v1.2rev57 with the “automated” parameter. The phylogenetic tree was constructed using the Maximum Likelihood method in the MEGA X software. The support for each node was tested with standard bootstrap analysis through 1,000 replications. The phylogenetic tree was visualized in iTOL v7.^5^

The feature of Dicer proteins was analyzed online at Pfam,^6^ SMART^7^ and NCBI (see text footnote 4). The secondary and tertiary structures of the *Cr*DCL1-3 proteins were predicted using JPred: A Protein Secondary Structure Prediction Server^8^ and SWISS-MODEL,^9^ respectively.

### 2.3 RNA extraction and analyses

Total RNA and Small RNA were extracted using RNAiso Plus (TaKaRa, Japan) and RNAiso for Small RNA (TaKaRa, Japan), respectively, following the manufacturer’s instruction. RNA quality was evaluated using a NanoDrop 2000 Ultra Microvolume Spectrophotometer (Thermo, MA, United States), with the 260/A280 ratios of total RNAs and sRNAs were approximately 2.0 and 1.75, respectively. For cDNA synthesis, genomic DNA was removed and reverse transcription was performed using the PrimeScript™ RT Reagent Kit with gDNA Eraser (Perfect Real Time) (TaKaRa, Japan). Reverse transcription of sRNAs was performed with specific stem-loop primers (Supplementary Table S2). RT-qPCR was performed to detect RNA transcripts using PrimeScript™ RT-PCR (TaKaRa, Japan) on the ABI 7300 Real-Time PCR System (Framingham, MA, United States). The *ACTIN* gene and *U4* snoRNA served as internal controls for normalizing mRNA and sRNA expression levels (Wang et al., 2017). All RT-qPCR primers are listed in Supplementary Table S3. The relative gene expression levels were calculated using the 2^−△△CT^ method from three technical replicates.

The detected miRNA levels were further validated by Northern blot analysis. Total RNA was denatured at 70°C and separated by polyacrylamide gel electrophoresis using 0.5x TBE buffer. The RNA was then wet-transferred to a Hybond-NX nylon membrane (GE Healthcare) at a constant current of 0.3A for 50 min. The membrane was cross-linked with EDC cross linking solution at 65°C for 90 min. Following pre-hybridization with Hybridization buffer at 55°C for 40 min, 50 nmol/L miRNA probe and 25 nmol/L U4 probe were added for hybridization for 16 h. All reagents were prepared according to the methods described previously (Martinho et al., 2023). The membrane was washed with elution buffer (2x SSC and 0.1% SDS) and visualized using Procedure for Detection of Immobilized Nucleic Acids (Thermo, United States) and ChemiScope 3,300 Mini system (Clinx Science Instruments, China). The probes used for Northern blot were biotin-labeled at both the 5 “and 3” ends, and their sequences were listed in Supplementary Table S4. Integrated density of the band in images was measured three times using imageJ software.

All experiments were performed with more than three biological replicates to ensure repeatability.

### 2.4 Small RNA-seq and analyses

#### 2.4.1 Sample preparation

After grown to the exponential phase (3.0–4.0 × 10^6^ cells ml^−1^), the algal cells were collected by centrifugation, stored in liquid nitrogen and sent to Gene Denovo Biotechnology Company (Guangzhou, China) for sRNA-seq and analyses. In total, there were two groups of samples (CC-5325 and *dcl1* mutant), each with three biological replicates.

#### 2.4.2 sRNA library construction and sequencing

Total RNAs were extracted by TRIzol, and 18–30 nt sRNAs were purified using polyacrylamide gel electrophoresis (PAGE), followed by 3′ and 5′ adapter ligation. The ligation products were reverse transcribed and amplified by PCR. The PCR products with the length of 140-160 bp were enriched to produce the cDNA libraries. Sequencing was performed using an Illumina HiSeq™ 2,500.

#### 2.4.3 miRNA identification

To obtain clean tags, the raw reads were filtered to remove the following: low quality reads (Q value ≤20 or containing unknown nucleotides N), reads without 3′ adapters or containing 5′ adapters, reads shorter than 18 nt (not including adapters) and reads containing polyA in the sRNA fragments. All clean tags were aligned with sRNAs in the GenBank (Release 209.0) and Pfam (11.0) databases to identify and eliminate rRNA, snoRNA, snRNA, and tRNA. They were also compared with the *Chlamydomonas* genome (v5.6) to remove repeat sequences and some tags that were potentially fragments from mRNA degradation. The remaining clean tags were queried against the miRBase database (Release 21) to identify the previously reported *Chlamydomonas* miRNAs (cre-miRNA), and the conservative miRNAs were obtained by alignment with miRNAs from other species. All unannotated tags were aligned with the *Chlamydomonas* genome, and novel miRNA candidates were predicted using Mireap_v0.2 software based on genome positions, hairpin structures, DCL cutting site, free energy and parameters between the miRNA and miRNA* strands (Ding et al., 2021; Kang et al., 2021).

#### 2.4.4 Expression analysis of miRNAs

miRNA expression levels in CC-5325 and *dcl1* were normalized according to the following formula: Transcripts per million (TPM) = $\frac{\text{Actual miRNA number}}{\;\text{Total number of Clean Tags}}$ × 10^6^. Furthermore, the fold change, the size of between-group differences and the associated *p*-values were calculated as following: Fold change (fc) = $\frac{\text{Normalized expression of}\;dcl1\;\text{group}}{\text{Normalized expression of}\;CC-5325\;\text{group}}$, and the size of between-group differences = log_2_(fc). Significant differences in miRNA expression level were identified based on a fold change ≥1.5 and *p*-value <0.05.

Target prediction for differentially expressed miRNAs and functional analysis.

Patmatch (v1.2) software was used to predict the targets of the differentially expressed miRNAs in the *Chlamydomonas* genome. To infer the potential functions of the differentially expressed miRNAs and their targets, we performed gene ontology (GO) enrichment analysis and Kyoto Encyclopedia of Genes and Genomes (KEGG) pathway enrichment analysis. For both analyses, significant enrichment was identified based on *p* < 0.05.

### 2.5 Statistical analysis

For RT-qPCR and Northern blot data, statistical significance was assessed using Dunnett’s multiple comparisons test and GraphPad Prism 9.1.2, at the levels of 0.01, 0.05, and 0.001. The data represents mean values from at least three biological replicates.

## 3 Results

### 3.1 Features of *Cr*DCL1-3 protein

**Figure 1:**
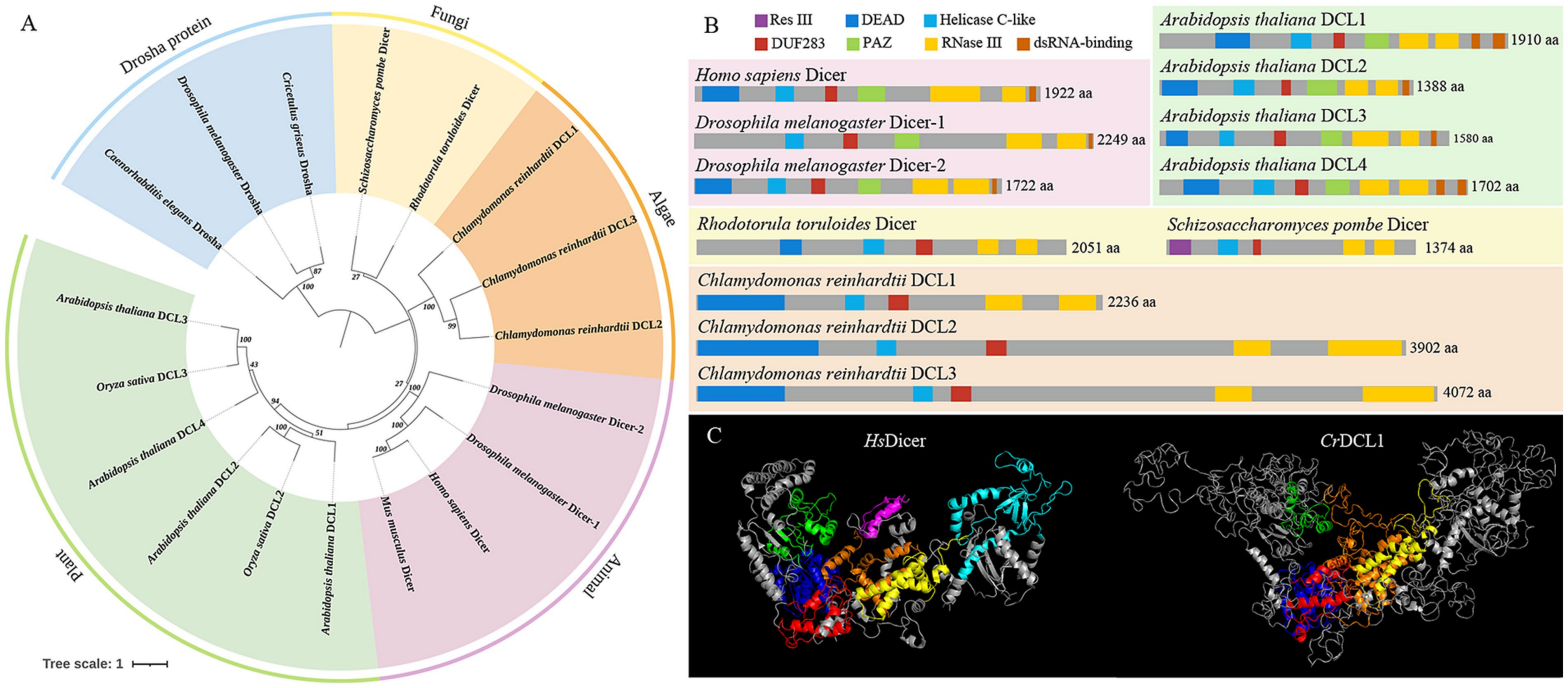
Feature of Dicer homologous proteins. **(A)** Phylogenetic tree analysis of Dicer and Drosha homologous proteins. **(B)** Structural domain analysis of Dicer homologous proteins. **(C)** Prediction of the three-dimensional structures of *Hs*Dicer and *Cr*DCL1 proteins. In the three-dimensional structure diagram, the dark blue structures are the DEAD domains, the green structures are the Helicase C-like domains, the red structures are the DUF283 domains, the light blue structure is the PAZ domain, the yellow structures are the first RNase III domains, the orange structures are the second RNase III domains, and the purple structure is the dsRNA-binding domain.

The Dicer proteins of *C. reinhardtii*, *A. thaliana*, *O. sativa*, *M. musculus*, *H. sapiens*, *D. melanogaster*, *R. toruloides*, and *S. pombe* were used to construct a phylogenetic tree in conjunction with the Drosha proteins of *C. griseus*, *D. melanogaster*, and *C. elegans* (Figure 1A). The analysis revealed that Drosha proteins of *C. griseus*, *D. melanogaster*, and *C. elegans* formed an outgroup, constituting a separate branch distinct from the Dicer proteins in each species. The Dicer/DCL proteins clustered according to their taxonomy categories: plants, animals, algae, and fungi. Within the plant branch, both *A. thaliana* and *O. sativa* exhibited multiple DCL proteins, which were grouped based on sequence similarity and function rather than species. For instance, *At*DCL2 and *Os*DCL2 clustered together, as did *At*DCL3 and *Os*DCL3. Three DCL proteins of *C. reinhardtii* formed an independent branch, evolutionarily distant from the Dicer/DCL proteins of other species.

The domain analyses of Dicer proteins revealed that *At*DCL1-4, *Hs*Dicer and *Dm*Dicer1-2 all possess a DEAD/H-box helicase (hereinafter referred to as DEAD), Helicase C-like, DUF283, PAZ domain, two RNase III domains, and one or two dsRNA-binding domains. In contrast, *Rt*Dicer, *Sp*Dicer and *Cr*DCL1-3 contain a DEAD, Helicase C-like, DUF283 and two RNase III domains but lack both PAZ and dsRNA-binding domains (Figure 1B). These findings suggested that DEAD, Helicase C-like, DUF283, and two RNase III domains are conserved across Dicer/DCL proteins, while PAZ and dsRNA-binding domains may have evolved later. Notably, *Sp*Dicer lacks the conserved DEAD domain but exhibits an additional unique Res III domain. There are not many reports on the Res III domain, and it remains unclear whether this domain compensates for the function of the DEAD domain or performs other unique functions.

Furthermore, *Cr*DCL1 is comparable to other Dicer proteins in length, but *Cr*DCL2 and *Cr*DCL3 has twice number of amino acids as others. Notably, attempts to predict the secondary structure of *Cr*DCL2 and *Cr*DCL3 were unsuccessful. Similarly, predictions of their three-dimensional structures remained incomplete. Only the conserved domains (DEAD, Helicase C-like, DUF283 domains, and two RNase III domains) could be simulated. The three-dimensional structure of *Cr*DCL1 and *Hs*Dicer was compared and results revealed an overall L-shaped structure for both proteins, with similar distribution patterns of their DEAD, Helicase C-like, DUF283 domains, and two RNase III domains. In this L-shaped structure, the Helicase C-like domains were located at the short arm, DEAD and DUF283 domains were located at the junction of the short and long arms, and two RNase III domains were located near DUF283 domain on the long arm. Additionally, the PAZ domain of *Hs*Dicer was located at the distal end of the long arm. It is important to note that *Cr*DCL1 lacks the PAZ domain and dsRNA-binding domain but exhibits numerous irregular curls (Figure 1C).

### 3.2 Screening and verification of the *dcl1* and *dcl3* mutants

The *dcl* mutants used in the present study were obtained from the *Chlamydomonas* Library Project (CLiP). Based on the recording, the *dcl1* mutant strains LMJ.RY0402.124662 (*dcl1*-124662) and LMJ.RY0402.198146 (*dcl1*-198146) from CLiP harbored the cassette in a CDS region and intron region of the *CrDCL1* gene, respectively. The *dcl3* strains LMJ.RY0402.080558 (*dcl3*-080558) and LMJ.RY0402.253048 (*dcl3*-253048) harbored the cassette in an intron region and CDS region of the *CrDCL3* gene, respectively.

**Figure 2:**
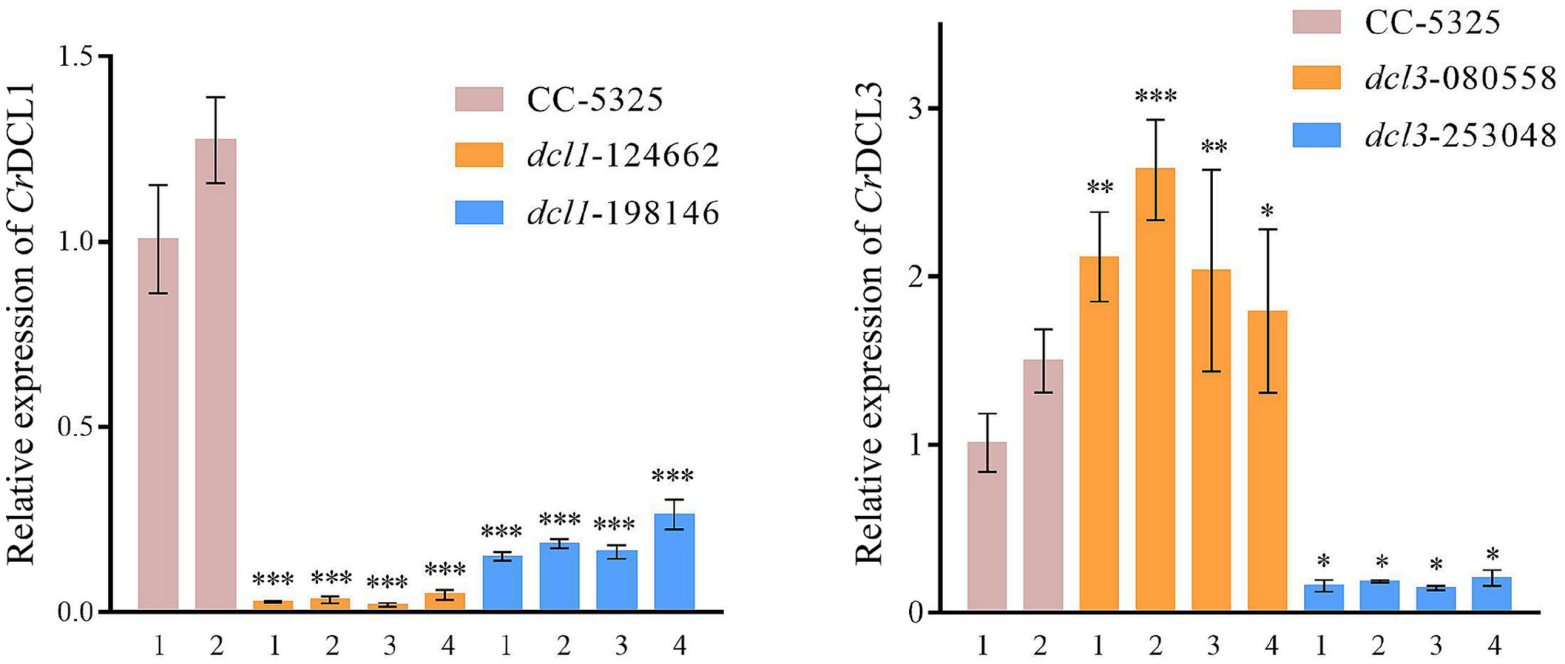
RT-qPCR analysis of *CrDCL1* and *CrDCL3* transcript levels in the mutants and control. *ACTIN* was used as an internal control for normalization. Bars indicate the standard error of the means (*n* = 3). *, **, and *** indicates the statistical significance between two means at the level of 0.05, 0.01, and 0.001, respectively.

We randomly selected four colonies of each mutant to confirm the DNA cassette insertion in the target gene, and then detected the mRNA transcript levels by RT-qPCR. The analysis showed that *CrDCL1* transcript levels were significantly down-regulated in *dcl1*-124662 and *dcl1*-198146; *CrDCL3* was significantly down-regulated in *dcl3*-253048 but increased in *dcl3*-080558 (Figure 2). Hence, for both genes, insertion of the cassette into the CDS region led to the significant inhibitory of *CrDCL1* and *CrDCL3* expression. *CrDCL1* and *CrDCL3* transcript levels were lowest in *dcl1*-124662 colony #3 and *dcl3*-253048 colony #3, respectively. Therefore, these two colonies were selected as the *dcl1* and *dcl3* mutants for the subsequent experiments.

Moreover, the expression of *CrDCL1* gene in *dcl3* mutant was no reduced, whereas *CrDCL3* transcripts were significantly increased in *dcl1* mutant (Supplementary Figure S1). This suggests that some functions of the *Cr*DCL1 protein overlap with those of the *Cr*DCL3 protein. In the *dcl1* mutant, the loss of *Cr*DCL1 protein function may lead to compensatory upregulation of *Cr*DCL3 protein expression to perform related tasks.

The expression levels of previously reported miRNAs were analyzed by Northern blot in the *dcl1* and *dcl3* mutants. miR1162, miR1151b and miRB were undetectable in *dcl3* (Supplementary Figure S2), indicating that *Cr*DCL3 protein mediates the biogenesis of these three miRNAs. This conclusion aligns with previous report (Valli et al., 2016). Notably, density analysis of the Northern blot bands revealed a slight downregulation of miR1162 and miR1151b in *dcl1* mutant relative to CC-5325, suggesting that *Cr*DCL1 protein may also play a role in the production of some miRNAs.

### 3.3 Analyses of CC-5325 and *dcl1* sRNA-seq data

**Figure 3:**
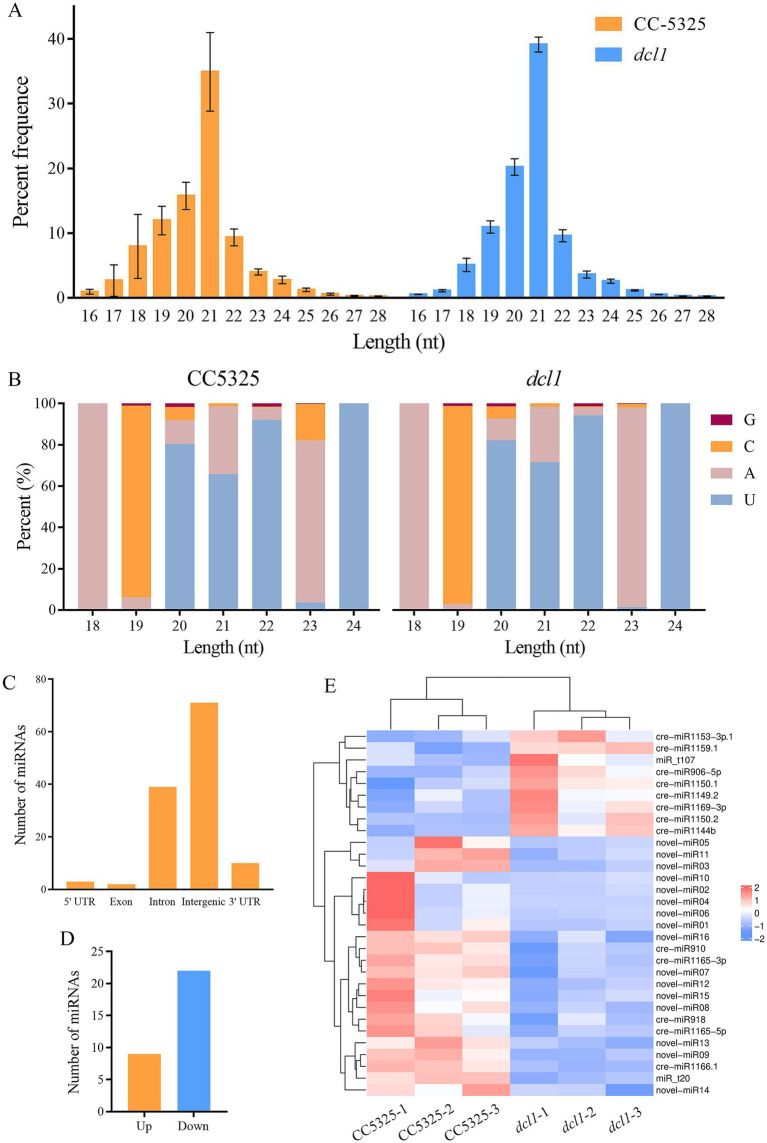
Characteristics of *C. reinhardtii* sRNAs from CC-5325 and the *dcl1* mutant. **(A)** Length distribution of sRNAs. **(B)** Nucleotide frequency at the 5′ end of all identified miRNAs. **(C)** Genome locations of all identified miRNAs. **(D)** The number of miRNAs with significantly up-regulated or down-regulated expression levels in *dcl1* compared to CC-5325. **(E)** Expression heat map of the differentially expressed miRNAs.

To investigate whether *Cr*DCL1 is involved in the biogenesis of some miRNAs in *C. reinhardtii*, we performed sRNA-seq for CC-5325 and *dcl1* mutant with three biological replicates. The sRNA characteristics were identical in the *dcl1* mutant and CC-5325, as previously reported (Zhao et al., 2007; Valli et al., 2016). The sRNAs were 16–28 nt long with a normal distribution, and 30–40% of the sRNAs were 21 nt long (Figure 3A).

By comparing the sequences in the miRBase database and previous reports (Shu and Hu, 2012; Valli et al., 2016; Yamasaki and Cerutti, 2017; Voshall et al., 2017; Zhang et al., 2021), 117 previously reported cre-miRNAs were obtained. Among them, 19 cre-miRNAs only record mature sequences and precursor sequences in the miRBase database, lacking information on their chromosomal location. The precursors of these 19 cre-miRNAs were aligned with the updated *Chlamydomonas* genome (v5.6) to obtain their chromosomal location information, but the match failed. Considering them as novel miRNAs, we further predicted the precursors of these 19 unmatched miRNAs. Results were as follows: 9 miRNAs had no corresponding precursor sequences in *C. reinhardtii*, 7 miRNAs had a corresponding precursor, and the predicted precursor sequences of the remaining 3 miRNAs showed that their mature sequences completely complement their miRNA* (Table 1; Supplementary Figure S3). The miRNAs were mostly 20–22 nt in length with an obvious peak at 21 nt, and there was a bias toward U as the first nucleotide among the 20–22 nt miRNAs (Figure 3B). Analysis of the genomic location of these miRNAs showed that most of them derived from intergenic regions (Figure 3C).

**Table 1:** Information for corrected cre-miRNAs from the miRBase database.

miRNA					Stem-loop				
Group^a^	Name	Sequence	Length (nt)	Arm	Chromosome	Start	End	Strand	Location
I	cre-miR1143-3p	TTATTTGCCCGAAGGGGACGTCCT	24	3p	−				
	cre-miR1143-5p	AGGACGTCCCCTTACGGGA	19	5p	−				
	cre-miR1144a.1	CAGGCAGCGCGGGGCTGCTGG	21	5p	−				
	cre-miR1144a.2	TGGAACCGGGCACGCAGGAG	20	5p	−				
	cre-miR1146	ATGGGTCCGATCGGGAAGCT	20	5p	−				
	cre-miR1148.1	CCAACGTGCAGGGGGACATGG	21	5p	−				
	cre-miR1158	ACTTGGAGGAGGCCACTGGC	20	3p	−				
	cre-miR909.1	TGCTGGTCAAACCGGTGGTGG	21	5p	−				
	cre-miR909.3	TTCAGGGTCAAGTTTGCATGC	21	3p	−				
II	cre-miR1145.2	TGGCGTTGACCCTGTCGGTGG	21	3p	Chromosome 13	552,482	552,630	+	3’UTR
	cre-miR1159.1	TGCCACAGTGCCCGATTGCCG	21	3p	Chromosome 14	1,191,546	1,191,789	−	Intron
	cre-miR909.2	ATGCAAACATGACCCTGAATG	21	3p	Chromosome 16	3,785,651	3,785,955	+	Exon
	cre-miR1165-3p	ACGGACCGCTTGTACGGATATG	22	3p	Chromosome 3	1,999,966	2,000,090	−	Intron
	cre-miR1165-5p	TACCGTACAAGCGGTCCGTCC	21	5p	Chromosome 3	1,999,966	2,000,090	−	Intron
	cre-miR1144b	TGGGTAGTGTGGCGGCAGGCAG	22	5p	Chromosome 4	619,405	619,644	+	Exon
	cre-miR1145.1	TTGGGGCCCAGCAGGTCCTGG	21	3p	Chromosome 13	3,793,072	3,793,434	+	Intergenic
III	cre-miR1159.2	ACAATGCCAATGGAGACGGAT	21	5p	Chromosome 14	1,191,574	1,191,761	+	Intron
	cre-miR1148.2	TGGAGATCCTCCTGTCCGGCT	21	5p	Chromosome 11	1,442,280	1,442,372	+	Intron
					Chromosome 11	1,442,279	1,442,371	−	Intron
	cre-miR907	TCTTCTGCGAGCGGTGCGAGC	21	3p	Chromosome 6	4,031,324	4,031,506	+	Exon
					Chromosome 6	4,031,325	4,031,507	−	Exon
					Chromosome 6	4,088,541	4,088,724	+	Exon
					Chromosome 6	4,088,542	4,088,725	−	Exon

a These miRNAs are classified into three classes: class I miRNAs have no corresponding precursor sequences, class II miRNAs have a precursor, class III miRNAs were considered to be siRNAs because of the mature sequences of these were completely complementary to their miRNA*..

### 3.4 *Cr*DCL1 is involved in miRNA biogenesis

**Figure 4:**
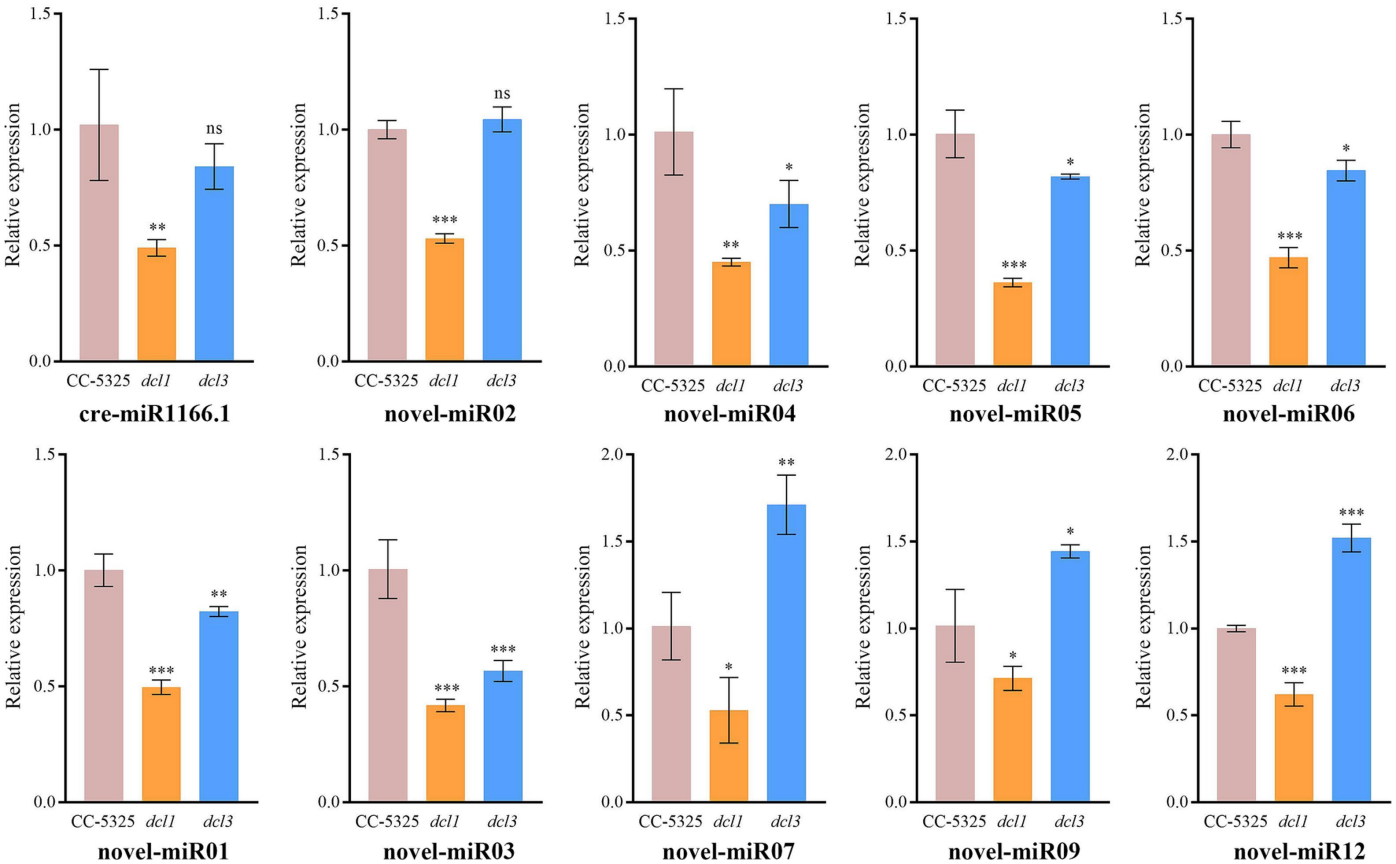
RT-qPCR analysis of 10 differentially expressed miRNAs in CC-5325, *dcl1* and *dcl3*. The *U4* gene was used for normalization. Error bars show the standard error from three technical replicates. ns, not significant; *p < 0.05; **p < 0.01; ***p < 0.001.

To identify miRNAs associated with *Cr*DCL1 from the sequencing data, we compared the miRNA expression levels in CC-5325 and *dcl1* mutant. The analysis identified 9 miRNAs with significantly increased levels in *dcl1* mutant [log_2_(fc) ≥ log_2_1.5, *p* < 0.05] and 22 miRNAs with significantly decreased levels [log_2_(fc) ≤ −log_2_1.5, *p* < 0.05], in comparison with CC-5325 (Figures 3D,E), suggesting that *Cr*DCL1 may be involved in the biogenesis of these 22 down-regulated miRNAs. Based on their expression fold change, 14 of these 22 miRNAs were classified as “high-differential miRNAs” [log_2_(fc) ≤ −1], and the remaining 8 were referred to as “medium-differential miRNAs” [−1 ≤ log_2_(fc) ≤ −log_2_1.5] (Table 2). These 14 high-differential miRNAs included one previously reported cre-miRNA (cre-miR1166.1) and 13 novel miRNAs. Among them, novel-miR02 had 3 precursors. The 8 medium-differential miRNAs included 4 previously reported cre-miRNAs and 4 novel miRNAs. Among them, the precursors of cre-miR1165-5p and cre-miR1165-3p were predicted again in this study (Table 1). To further investigate the potential involvement of *Cr*DCL1 in the biogenesis of these miRNAs, we examined the expression levels of 10 high-differential miRNAs using RT-qPCR. The results showed that the expression levels of these 10 high-differential miRNAs were significantly down-regulated in *dcl1* compared with CC-5325 (Figure 4). These results were consistent with the sRNA-seq data, which not only served as evidence for the accuracy of sRNA-seq data, but also proved that *Cr*DCL1 protein affected the production of these 10 high-differential miRNAs. We also detected the expression levels of these miRNAs in the *dcl3* mutant. Interestingly, the abundances of 5 novel miRNAs (novel-miR01, novel-miR03, novel-miR04, novel-miR05, and novel-miR06) in the *dcl3* mutant were lower than that in CC-5325 but higher than that in *dcl1*, whereas three novel miRNAs (novel-miR07, novel-miR09, and novel-miR12) were up-regulated in *dcl3* mutant relative to CC-5325 (Figure 4), indicating that the production of some miRNAs was mediated by *Cr*DCL1 and *Cr*DCL3 proteins.

**Table 2:** Down-regulated miRNAs in the *dcl1* mutant.

miRNA				Stem-loop				Quantification^b^		
Group^a^	ID	Length (nt)	Sequence	Chromosome	Start	End	Location	CC-5325	*dcl1*	log_2_(fc)
High-differential miRNAs	novel-miR01	21	AATTACCTATCATTCGTGGGT	Chromosome 5	2,873,872	2,874,135	Intergenic	3.5	0.15	−4.523
	novel-miR02	21	TGGGTCACCTGCGCCTGCGTT	Chromosome 14	2,122,168	2,122,460	Intergenic	1.37	0.08	−4.169
				Chromosome 14	2,142,687	2,142,961	Intron			
				Chromosome 14	2,148,721	2,148,995	Intron			
	novel-miR03	23	TGAATGTAAACTCCCCCTCCCCA	chromosome_11	2,785,306	2,785,459	Intron	1.18	0.08	−3.956
	novel-miR04	23	CAGCGGTGGGCTGAGGGTAGACG	Chromosome 12	8,503,745	8,503,841	Intron	375.57	32.48	−3.531
	novel-miR05	20	TATGCTGAGCACCCCGGTCG	Chromosome 10	3,368,596	3,368,743	Intergenic	3.11	0.32	−3.289
	novel-miR06	23	TACGCATCCTAAGTCGAGTCGTG	Chromosome 12	9,061,162	9,061,249	Intron	55.04	6.52	−3.078
	cre-miR1166.1	21	TGGACCTCGCGGCCCTGGAGG	Chromosome 5	577,911	578,282	Intergenic	14.93	1.83	−3.028
	novel-miR07	20	TGCGGTCGGCGTGTGTGTGG	Chromosome 10	1,120,701	1,121,049	Intron	2.62	0.67	−1.976
	novel-miR08	21	AACAGGTTATGAGCCCCGGAC	Chromosome 7	2,371,050	2,371,155	Intron	3.22	0.85	−1.92
	novel-miR09	21	ACGCCGATGAACTCTGCAATG	Chromosome 13	2,605,312	2,605,488	Intergenic	4.13	1.24	−1.737
	novel-miR10	20	CCCGTTCCACTGGGACATCC	Chromosome 12	6,266,530	6,266,662	Intron	61.72	19.9	−1.633
	novel-miR11	20	TAGGATCCTAATGAATGTGA	Chromosome 1	7,867,220	7,867,479	Intron	5.14	1.98	−1.376
	novel-miR12	23	ACCGGTCGAGAGAGTGTTGTCGG	Chromosome 15	299,894	300,127	Intron	31.24	12.49	−1.322
	novel-miR13	21	TAGCCAACAAGGCCGCCGAAG	Chromosome 15	703,183	703,279	Intron	9.78	4.3	−1.184
Medium-differential miRNAs	cre-miR1165-5p	21	TACCGTACAAGCGGTCCGTCC	Chromosome 3	1,999,966	2,000,090	Intron	2745.53	1478.23	−0.893
	novel-miR14	22	TTAGGCCCCGTGCTGGCGAATG	Chromosome 17	1,131,227	1,131,348	Intron	11.19	6.12	−0.87
	cre-miR1165-3p	22	ACGGACCGCTTGTACGGATATG	Chromosome 3	1,999,966	2,000,090	Intron	3202.79	1831.38	−0.806
	novel-miR15	21	AGGCCTATTACCGCGTCGAAG	Chromosome 7	2,713,511	2,713,622	Intron	19.31	11.05	−0.806
	cre-miR918	21	TACCTGAAGCGGACATCTTGC	Chromosome 5	1,014,118	1,014,386	Intergenic	221.83	130.13	−0.769
	cre-miR910	21	AGCAGCGTCGGGCTCGACCGC	Chromosome 14	966,108	966,334	Intergenic	40883.05	24703.94	−0.727
	novel-miR16	21	TAGCCGATGGAACCCCCAGCT	Chromosome 5	910,438	910,438	Intron	16175.43	9927.71	−0.704
	miR_t20*	21	TAGAGCTCGAAGAACTTGGGA	Chromosome 6	6,776,096	6,776,203	Intergenic	98402.4	62535.6	−0.654

a The down-regulated miRNAs are classified as “high-differential miRNAs” with log_2_(fc) ≤ −1 and “medium-differential miRNAs” with −1 ≤ log_2_(fc) ≤ −log_2_1.5 (*p* < 0.05).

b Average of normalized reads from three independent libraries (*n* = 3).

^*^The name for previously identified miRNA was taken from Voshall et al. (2017).

We also performed Northern blot analysis to further validate the expression levels of the high-differential miRNAs in the mutants and control. However, due to their extremely low expression levels, only one of these high-differential miRNAs (novel-miR04) was detectable by Northern blot. Consisted with the RT-qPCR result, the abundance of novel-miR04 was slightly reduced in *dcl3* and significantly reduced in *dcl1* compared to the control (Supplementary Figure S4). Thereby, it strongly proposed that *Cr*DCL1 is involved in the biogenesis of novel-miR04. We also analyzed the expression levels of cre-miR910, a confirmed cre-miRNA. Northern blot analysis indicated that cre-miR910 was significantly down-regulated in *dcl1* and reduced to an undetectable level in *dcl3* (Supplementary Figure S4). These results suggest that *Cr*DCL1 and *Cr*DCL3 are both involved in the biogenesis of some miRNAs (e.g., novel-miR04 and cre-miR910).

### 3.5 Target gene prediction of the down-regulated miRNAs and functional analysis

**Figure 5:**
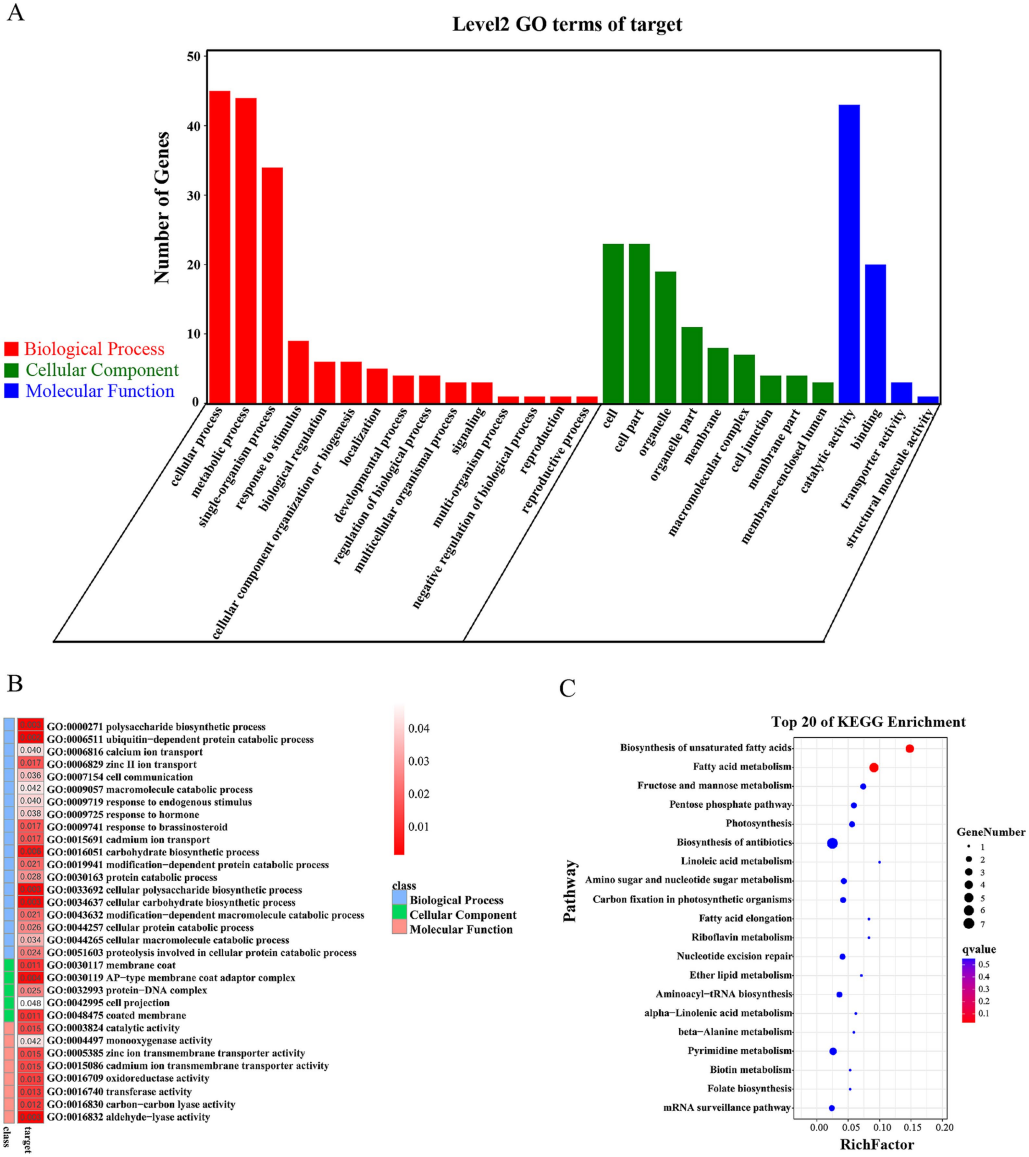
Functional analysis of the predicted targets of *Cr*DCL1-related miRNAs. **(A)** Categorization of miRNA target genes by GO analysis. **(B)** Analysis of the miRNA target genes revealed a significant enrichment of various molecular function, cellular component and biological process terms. **(C)** KEGG pathway enrichment analysis of the predicted miRNA targets.

Finally, we performed target gene prediction and functional analysis for the 22 down-regulated miRNAs. A total of 384 target genes were obtained, 105 of which were target genes of previously reported cre-miRNAs. GO analysis was performed for all target genes, and the results indicated their involvement in various cellular components and molecular functions such as catalytic activity and binding. These target genes also have important roles in many biological processes including cellular processes, metabolic processes and single-organism processes (Figure 5A). The following cellular component terms were significantly enriched in the dataset, with 26 target genes associated with AP-type membrane coat adaptor complex, membrane coat, coated membrane, protein-DNA complex and cell projection. Forty-nine target genes were associated with lyase activity, transferase activity, oxidoreductase activity, catalytic activity, ion transmembrane transporter activity and monooxygenase activity (Figure 5B). KEGG pathway enrichment analysis was also performed. Among 40 target genes with functional descriptions associated with metabolic and signal transduction pathways, there was a significant enrichment of genes associated with fatty acid metabolism and the biosynthesis of unsaturated fatty acids (Figure 5C).

Analysis of genes and miRNAs related to the fatty acid metabolic pathway revealed that the *Cre03.g213313*, *Cre04.g217945*, and *Cre01.g035400* genes had significant differences in the fatty acid metabolic pathway. Among them, *Cre03.g213313* and *Cre04.g217945* were involved in the unsaturated fatty acid biosynthesis pathway (Table 3). Target gene prediction revealed that *Cre03.g213313*, functioning as 3-oxoacyl-(fabG) is the target gene of novel-miR16; *Cre04.g217945*, functioning as a fatty acid desaturase (such as SCD, desC, stearoyl-CoA desaturase) is the target gene of novel-miR07; and *Cre01.g035400*, functioning as an E3 ubiquitin ligase (MECR, NRBF1; mitochondrial trans-2-enoyl-CoA reductase), is the target gene of novel-miR02 (Table 3).

**Table 3:** Gene information enriched in fatty acid metabolic pathways.

miRNA	Predicted target gene	Description	Metabolic pathway
novel-miR16	Cre03.g213313	3-oxoacyl-(fabG)	Biosynthesis of unsaturated fatty acids, Fatty acid metabolism.
novel- miR07	Cre04.g217945	SCD, desC; stearoyl-CoA desaturase (Delta-9 desaturase)	
novel- miR04	Cre01.g035400	MECR, NRBF1; mitochondrial trans-2-enoyl-CoA reductase	Fatty acid metabolism

## 4 Discussion

### 4.1 Nineteen cre-miRNAs from the miRBase database were corrected in this study

In 2007, both Zhao et al. and Molnár et al. validated the miRNAs in *C. reinhardtii* identified in their studies, by alignment with the *Chlamydomonas* genome (v3.0) (Zhao et al., 2007; Molnár et al., 2007). Currently, 137 cre-miRNAs are listed in the miRBase database, most of which derived from their studies (Zhao et al., 2007; Molnár et al., 2007). According to several recent studies, an updated version of the *Chlamydomonas* genome was available (v5.6). Based on this updated v5.6 version, we found that 19 cre-miRNA precursors from miRBase were incorrect, and we re-predicted the precursors of these miRNAs. Our analysis indicates that 9 of these cre-miRNAs without precursors should be classified as non-miRNAs, 3 of these cre-miRNAs were completely complementary to their miRNA* and should be classified as siRNA, and the remaining 7 cre-miRNAs with a precursor are miRNA candidates.

### 4.2 Functional analysis of *Cr*DCL protein domains

Current studies have shown that fungi and most animals have only a single Dicer protein (Zhang et al., 2004; Drinnenberg et al., 2009; Makino et al., 2015), a few invertebrates have two Dicer proteins (Gao et al., 2014). Plants have multiple Dicer-like proteins (DCL) (Yu et al., 2017), and *C. reinhardtii* encodes three DCL proteins (*Cr*DCL1-3) (Lou et al., 2018). We performed phylogenetic analysis using Dicer/DCL proteins from multiple species and found that the *Cr*DCLs proteins formed an independently branch, and lacked PAZ and dsRNA-binding domains like *Rt*Dicer and *Sp*Dicer. In plants and animals, the PAZ and dsRNA-binding domains of Dicer/DCL proteins primarily influence their binding affinity for dsRNA. Moreover, the PAZ domain recognizes the 3′ end of pre-miRNA, serving as the starting point for Dicer/DCL cleavage, while the two RNase III domains function as a molecular ruler, enabling precise cutting of small RNAs into lengths of 21–25 nucleotides (Zhang et al., 2004; Macrae et al., 2006; Song and Rossi, 2017). Therefore, we speculate that Dicer/DCL proteins of animals and plants evolved from ancestral forms lacking PAZ and dsRNA-binding domains (such as *Cr*DCL and *Rt*Dicer), leading to more accurate and specific RNA binding.

Previous studies have shown that the feature of the DUF283 domain of *At*DCL4 and human Dicer protein are similar to that of the dsRNA-binding domain, and *At*DCL4 DUF283 domain has a weak binding ability to dsRNA (Qin et al., 2010; Liu et al., 2018); human Dicer DUF283 domain can bind single-stranded nucleic acids *in vitro* (Kurzynska-Kokorniak et al., 2016). Our laboratory has also preliminarily proved that the DUF283 domain of the *Cr*DCL protein can bind to dsRNA in vitro, through fluorescence anisotropy experiments (unpublished). On the other hand, DUF283 domain can recruit other dsRNA-binding domain proteins and participate in the binding of Dicer partner proteins (Dlakić, 2006). Therefore, we speculate that the DUF283 domain of the *Cr*DCL protein may replace or recruit other proteins to replace partial functions of the PAZ and dsRNA-binding domains. Besides, previous studies have reconstructed the 3D structure of *Hs*Dicer protein, showing the overall L-shaped structure (Lau et al., 2009; Paturi and Deshmukh, 2021). The three-dimensional structure of the *Cr*DCL1 protein predicted in this study is similar to that of the *Hs*Dicer protein. The distribution of various domains in *Cr*DCL1 and *Hs*Dicer are similar. However, *Cr*DCL1 has numerous irregular curls, whether they can replace the function of the PAZ and dsRNA-binding domain is still unclear and further verification needs to be conducted.

Since the coding sequences of *Cr*DCL1 and *Cr*DCL3 proteins are too long and have high GC content, it is a big challenge for full gene cloning. No signal peptide was predicted in *Cr*DCL1 and *Cr*DCL3, but the DEAD domains was presented and involved in various aspects of RNA metabolism, such as nuclear transcription, pre-mRNA splicing and nucleocytoplasmic transport (Schutz et al., 2010; Paysan-Lafosse et al., 2023). Therefore, we performed subcellular localization analysis using the 5′ partial (including the DEAD domain) of *Cr*DCL1 and *Cr*DCL3 proteins and found that they were both localized in the nucleus of onions (unpublished), which is consistent with the localization of *Cr*DUS16 protein. It is preliminarily believed that, like plants, *Cr*DCL proteins perform cleavage in the nucleus.

### 4.3 miRNA biogenesis in *C. reinhardtii* differs from that in animals and plants

*Cr*DCL3 is one of the three *Cr*DCL proteins (*Cr*DCL1-3) encoded in the *C. reinhardtii* genome, and it is mainly involved in miRNA biogenesis (Valli et al., 2016). *Cr*DCL1 mediates siRNA biogenesis (Casas-Mollano et al., 2008), but it was not previously reported associating with miRNA biogenesis. *Cr*DCL2 is not well characterized. In our analysis, most of the analyzed miRNAs were not detected in the *dcl3* mutant by Northern blot. In addition, 9 miRNAs were detected to be significantly up-regulated in the *dcl1* mutant by sRNA-seq. Combined with the result of up-regulated *Cr*DCL3 protein expression in the *dcl1* mutant, it can be speculated that the production of these 9 miRNAs may be affected by *Cr*DCL3 protein rather than *Cr*DCL1. This finding aligns with the established role of *Cr*DCL3 in miRNA biogenesis. Interestingly, sRNA-seq identified 22 miRNAs significantly down-regulated in the *dcl1* mutant compared to the control. Validation of the sRNA-seq data by RT-qPCR and Northern blot provided further evidence showing that *Cr*DCL1 is involved in the biogenesis of these 22 miRNAs.

In animals, only one Dicer protein is expressed, and miRNAs are generated by two cleavage steps performed by Drosha and Dicer, respectively (Zhao et al., 2007). In plants, multiple DCL proteins are presented, but only one DCL protein involved in miRNA biogenesis. For instance, most miRNAs in *Arabidopsis* are generated only depend on *At*DCL1 cleavage, while miR822, miR839 and miR859 only depend on *At*DCL4 rather than *At*DCL1 (Kurihara and Watanabe, 2004; Rajagopalan et al., 2006; Yu et al., 2017). Noticeably, 22 *Cr*DCL1-related miRNAs (down-regulated miRNAs in *dcl1* relative to the control) were still partially expressed in the *dcl1* mutant. The RT-qPCR and Northern blot results of this study showed that cre-miR910, novel-miR01, novel-miR03, novel-miR04, novel-miR05 and novel-miR06 were down-regulated at different degrees in the *dcl1* and *dcl3* mutant, suggesting that along with *Cr*DCL1, *Cr*DCL3 plays a key role in the biogenesis of these miRNAs. It is concluded the biogenesis of miRNAs in *C. reinhardtii* may be performed by multiple *Cr*DCL proteins, which is different from animals and plants. Nevertheless, the mechanisms of that remained to be explored.

Additionally, among the 10 high-differential miRNAs were detected by RT-qPCR, only 5 miRNAs were down-regulated in *dcl3* mutant, and the down-regulation amplitude was smaller than that in the *dcl1* mutant; compared with the control, the expression of *CrDCL1* gene showed no difference in *dcl3* mutants, whereas the *CrDCL3* gene were significantly increased in *dcl1* mutants. These results suggested that the production of these high-differential miRNAs mainly relied on the regulation of *Cr*DCL0031 protein, but the function of *Cr*DCL1 on the biogenesis of some miRNAs could be replaced by *Cr*DCL3 in *dcl1* mutants, thereby ensuring that algal cells can continue to produce these miRNAs. Whether this type of miRNA has important functions such as maintaining normal cell growth remains to be explored. Cre-miR910, as a medium-difference miRNA, was significantly down-regulated in *dcl1* and undetectable in *dcl3* compared to control. It is speculated that the medium-differential miRNAs were mainly relied on the cleavage of *Cr*DCL3 protein, but this speculation still needs more verification.

Finally, we performed preliminarily prediction and analysis of the biological metabolic pathways potentially mediated by *Cr*DCL1 related miRNAs. This study aims to provide a reference for further research on the mechanisms and biotechnological applications of *Cr*DCL1 protein and its related miRNA-mediated metabolic pathways. GO enrichment analysis indicated that the predicted target genes of these 22 miRNAs are associated with various molecular functions, cellular components and biological processes. Pathway enrichment analysis of their predicted target genes showed a significant enrichment of genes involved infatty acid metabolism. However, further analysis of significantly enriched target genes revealed only three miRNAs (novel-miR16, novel-miR07, novel-miR02) were associated with fatty acid metabolism. Notably, the predicted target gene of novel-miR02 is *MECR*. Previous study has demonstrated that the expression of the *MECR* gene in *C. reinhardtii* can enhance the production of total lipids and astaxanthin heterologous content (Sun et al., 2023). Our RT-qPCR experiment indicated that the expression of novel-miR02 primarily depends on *Cr*DCL1 protein but not on *Cr*DCL3 protein. We conducted fatty acid content detection in *dcl1*, *dcl3* mutants as well as the control strain CC-5325 using the method described by Jia et al. (2019). By comparing with standards, 11 credible peaks were identified. The relative fatty acids contents were obtained using peak area normalization, and the average values from two biological replicates were calculated to obtain the data presented in Supplementary Table S5. Results showed that the contents of 9,12,15-octadecatrienoic acid, methyl ester, (Z,Z,Z-) and methyl 4,7,10,13-hexadecatetraenoate in *dcl1* were slightly altered compared with CC-5325 (Supplementary Table S5). Therefore, whether novel-miR16, novel-miR07 and novel-miR02 are involved in the regulation of fatty acid metabolism, and whether the functional loss of *Cr*DCL1 protein leading to the downregulation of novel-miR02 increases *MECR* gene expression, thereby affecting total lipids and astaxanthin heterologous content, requires further verified.

## 5 Conclusion

In summary, the evolutionary trends and structural predictions of Dicer/DCL proteins across various species indicated that DEAD, Helicase C-like, DUF283, and two RNase III domains are conserved domains in Dicer/DCL proteins. In contrast, the PAZ and dsRNA-binding domains, absent in *Cr*DCL proteins, may have evolved later, contributing to the increased precision and complexity of small RNA generation in plants and animals. Moreover, based on the updated *Chlamydomonas* genome (v5.6), we excluded 12 miRNAs and re-predicted the precursors of 7 cre-miRNAs in miRBase. By analyzing miRNA expression levels, we demonstrated that *Cr*DCL1 is involved in the biogenesis of 22 miRNAs. Notably, the production of cre-miR910, novel-miR01, novel-miR03, novel-miR04, novel-miR05 and novel-miR06 were regulated not only by *Cr*DCL1 but also by *Cr*DCL3. These findings highlight that the production of some *C. reinhardtii* miRNAs may involve multiple *Cr*DCL proteins, differing from pathways observed in animals and plants. Furthermore, we performed target gene prediction analysis on 22 miRNAs and found that novel-miR02 may target the *MECR* gene, which is involved in the regulation of lipids and astaxanthin heterologous production. Despite these insights, there are still have many unresolved questions regarding the function of *Cr*DCL proteins and the biogenesis of miRNAs in *C. reinhardtii*, necessitating further investigation.

## Data Availability

Small RNA-seq datasets generated during this study have been submitted to the Genome Sequence Archive (GSA; https://ngdc.cncb.ac.cn/gsa/) under the accession number CRA023114.

## Footnotes

^1^ https://www.chlamylibrary.org/index

^2^ https://www.chlamylibrary.org/showCassette?cassette=CIB1

^3^ https://phytozome.jgi.doe.gov/pz/portal.html#

^4^ https://www.ncbi.nlm.nih.gov/

^5^ https://itol.embl.de/login.cgi?logout=1

^6^ http://pfam.xfam.org/

^7^ http://smart.embl-heidelberg.de/smart/set_mode.cgi?NORMAL=1

^8^ http://www.compbio.dundee.ac.uk/jpred4/index_up.html

^9^ https://swissmodel.expasy.org/interactive

## Supplementary material

Supplementary Figure S1 RT-qPCR analysis of *CrDCL1* and *CrDCL3* genes in CC-5325, *dcl1* and *dcl3*. *ACTIN* was used as an internal control for normalization. Bars indicate the standard error of the means (*n* = 3). ns, not significant; **p* < 0.05; ***p* < 0.01.

Supplementary Figure S2 Northern blot detection of miR1162, miR1151-b and miRB in the mutants and control. *U4* was used as an internal control.

Supplementary Figure S3 Predicted precursor structures of cre-miR1159.2, cre-miR1148.2, and cre-miR907.

Supplementary Figure S4 Northern blot detection of novel-miR04 and cre-miR910 in the mutants and control. *U4* gene was used as an internal control.

Supplementary Table S1 PCR primers for the *CrDCL* genes.

Supplementary Table S2 Specific stem-loop primers for reverse transcription.

Supplementary Table S3 RT-qPCR primers for miRNAs, *CrDCLs*, *ACTIN* and *U4*.

Supplementary Table S4 Northern blot probes for miRNAs and the *U4* gene.

Supplementary Table S5 Determination of the fatty acid content.
