# Supplementary figures and images for: Identification of CrDCL1-mediated microRNA biogenesis in green alga Chlamydomonas reinhardtii
Source: Front Microbiol. 2025 Feb 27;16:1487584. doi: 10.3389/fmicb.2025.1487584 (PMC11905391; doi:10.3389/fmicb.2025.1487584)

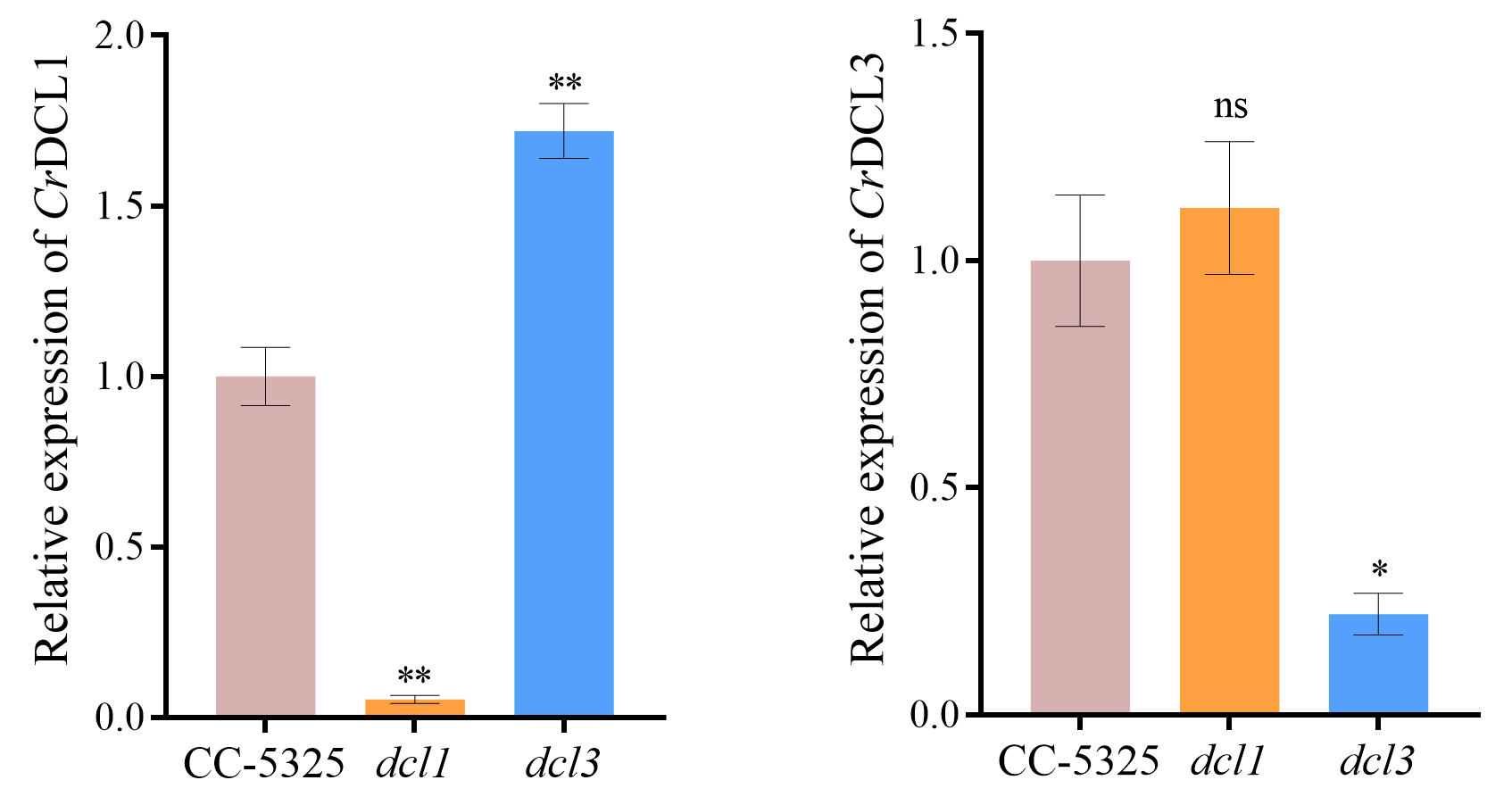

Supplement: Supplementary Figure S1 — RT-qPCR analysis of CrDCL1 and CrDCL3 genes in CC-5325, dcl1 and dcl3. ACTIN was used as an internal control for normalization. Bars indicate the standard error of the means (n = 3). ns, not significant; *p < 0.05; **p < 0.01. [file Image_1.tif]

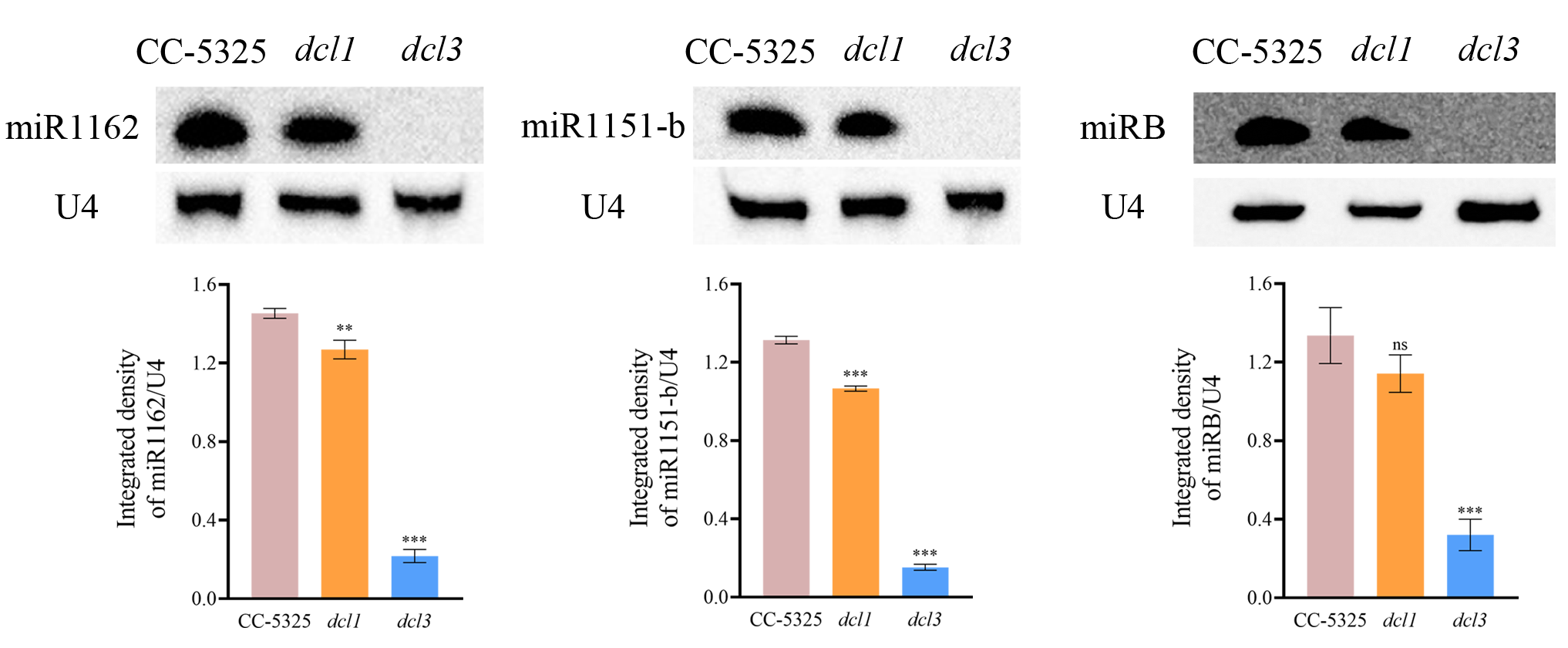

Supplement: Supplementary Figure S2 — Northern blot detection of miR1162, miR1151-b and miRB in the mutants and control. U4 was used as an internal control. [file Image_2.tif]

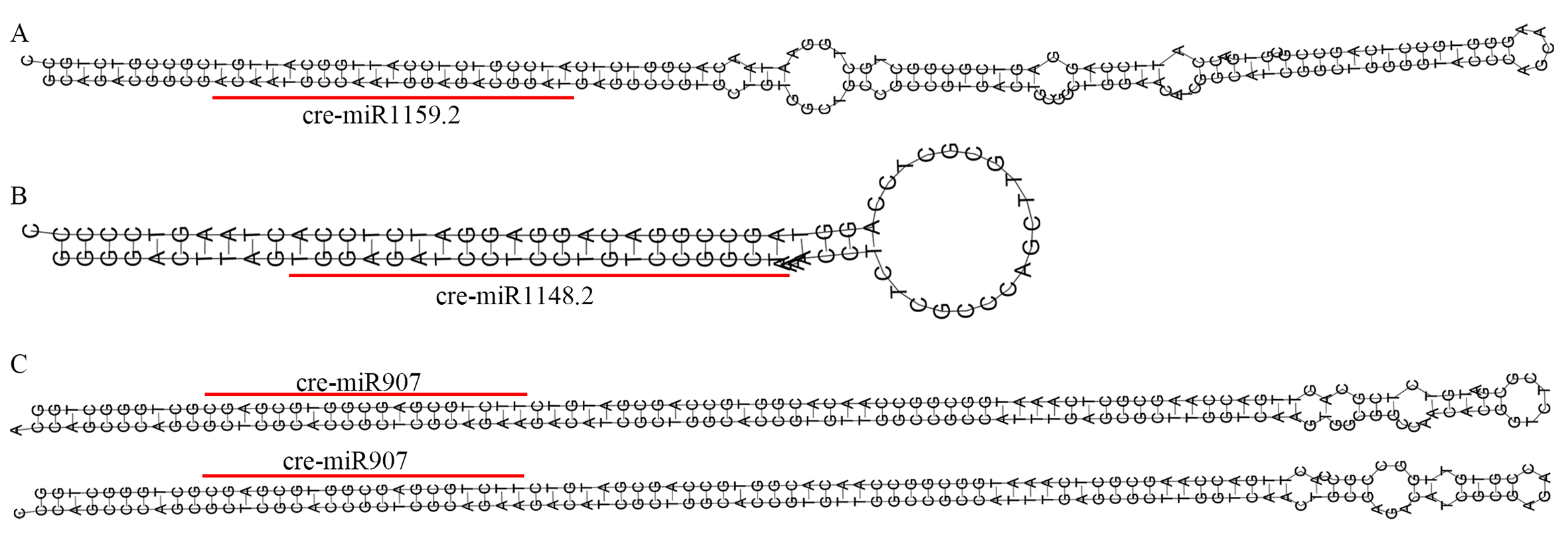

Supplement: Supplementary Figure S3 — Predicted precursor structures of cre-miR1159.2, cre-miR1148.2, and cre-miR907. [file Image_3.tif]

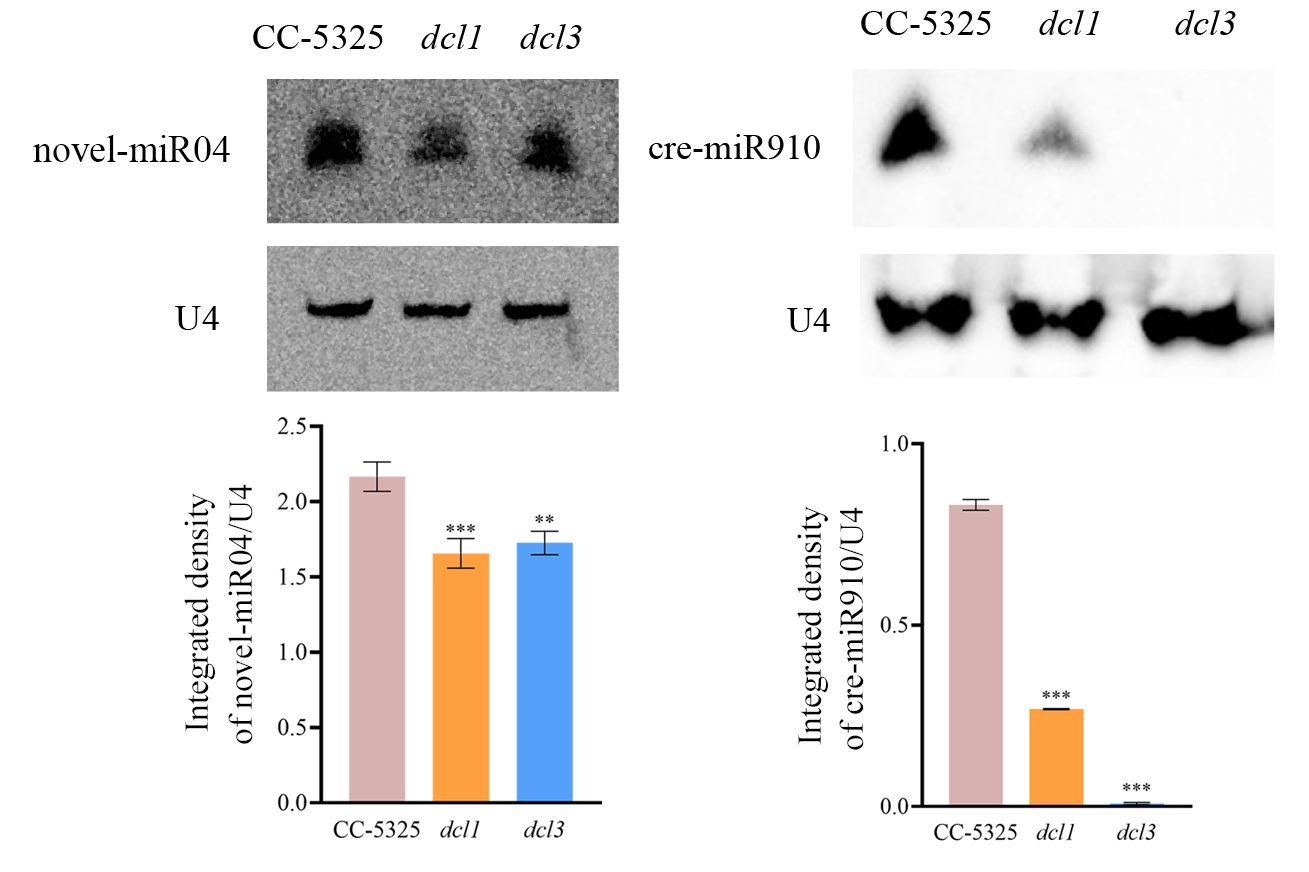

Supplement: Supplementary Figure S4 — Northern blot detection of novel-miR04 and cre-miR910 in the mutants and control. U4 gene was used as an internal control. [file Image_4.tif]
