# Supplementary material for: Identification of CrDCL1-mediated microRNA biogenesis in green alga Chlamydomonas reinhardtii
Source: Front Microbiol. 2025 Feb 27;16:1487584. doi: 10.3389/fmicb.2025.1487584 (PMC11905391; doi:10.3389/fmicb.2025.1487584)
Supplement: Supplementary file 5 [file Table_1.docx]

***Supplementary Material***

## Supplementary Tables

**Supplementary Table S1.** PCR primers for the *CrDCL* genes

| Primer name | Primer sequence (5’-3’) |
| --- | --- |
| DCL124662-F | CATGGAGTCGCTGCTAATCA |
| DCL124662-R | CATGGAGTCGCTGCTAATCA |
| DCL198146-F | TGTAGACACGGATCCAGCAG |
| DCL198146-R | ACACACACACACACACGCAC |
| DCL3-F | CCGTGTGTGTGTGTGTGTGT |
| DCL3-R | TTGTCGACGTTGACTTAGCG |

**Supplementary Table S2.** Specific stem-loop primers for reverse transcription

| Primer name | Primer sequence (5’-3’) |
| --- | --- |
| novel-miR0556-5p-RT | GGTCGTATGCAAAGCAGGGTCCGAGGTATCCATCGCACGCATCGCACTGCATACGACCacccac |
| novel-miR0183-3p-RT | GGTCGTATGCAAAGCAGGGTCCGAGGTATCCATCGCACGCATCGCACTGCATACGACCaacgca |
| novel-miR0075-5p-RT | GGTCGTATGCAAAGCAGGGTCCGAGGTATCCATCGCACGCATCGCACTGCATACGACCtgggag |
| novel-miR0128-5p-RT | GGTCGTATGCAAAGCAGGGTCCGAGGTATCCATCGCACGCATCGCACTGCATACGACCcgtcta |
| novel-miR0036-3p-RT | GGTCGTATGCAAAGCAGGGTCCGAGGTATCCATCGCACGCATCGCACTGCATACGACCcgaccg |
| novel-miR0102-5p-RT | GGTCGTATGCAAAGCAGGGTCCGAGGTATCCATCGCACGCATCGCACTGCATACGACCcacgac |
| cre-miR1166.1-RT | GGTCGTATGCAAAGCAGGGTCCGAGGTATCCATCGCACGCATCGCACTGCATACGACCccaggg |
| novel-miR0029-3p-RT | GGTCGTATGCAAAGCAGGGTCCGAGGTATCCATCGCACGCATCGCACTGCATACGACCccacac |
| novel-miR0155-5p-RT | GGTCGTATGCAAAGCAGGGTCCGAGGTATCCATCGCACGCATCGCACTGCATACGACCcattgc |
| novel-miR0193-3p-RT | GGTCGTATGCAAAGCAGGGTCCGAGGTATCCATCGCACGCATCGCACTGCATACGACCccgaca |

**Supplementary Table S3.** qRT-PCR primers for miRNAs, *CrDCL*s, *ACTIN* and *U4*

| Primer name | Primer sequence (5’-3’) |
| --- | --- |
| Actin F | ACCCCGTGCTGCTGACTG |
| Actin R | ACGTTGAAGGTCTCGAACA |
| DCL1-3’F | TGGAACACAGGCGAGGAGGC |
| DCL1-3’R | GTGGCAGAGACAGGGACAAG |
| DCL3-3’F | CCGGACGTGACGCTGGCATTC |
| DCL3-3’R | CGTGTCAGCACGCCATGGTG |
| U4 F | CAAAAGGCCCGACAGAAAT |
| U4 R | GTGAGGTCTAACCGAGTCGC |
| Universalreverse^a^ | GAGCAGGGTCCGAGGT |
| novel-miR0556-5p-F | AATTACCTATCATTCGTGGGT |
| novel-miR0183-3p-F | TGGGTCACCTGCGCCTGCGTT |
| novel-miR0075-5p-F | TGAATGTAAACTCCCCCTCCCCA |
| novel-miR0128-5p-F | CAGCGGTGGGCTGAGGGTAGACG |
| novel-miR0036-3p-F | TATGCTGAGCACCCCGGTCG |
| novel-miR0102-5p-F | TACGCATCCTAAGTCGAGTCGTG |
| cre-miR1166.1-F | ACCTCGCGGCCCTGG |
| novel-miR0029-3p-F | CCACACACACGCCGACCGCA |
| novel-miR0155-5p-F | CATTGCAGAGTTCATCGGCGT |
| novel-miR0193-3p-F | CCGACAACACTCTCTCGACCGGT |

^a^The reverse primer of miRNAs were all designed in the universal stem-loop.

**Supplementary Table S4.** Northern blot probes for miRNAs and the *U4* gene

| Probe name | Probe sequence (5’-3’) |
| --- | --- |
| U4-probe | GAAGAACGACGACCGACGGGGATGGAATAAGTTTTC |
| miR1151b-5p probe | TAACAGGTTATGAGCCCCGGA |
| miR1162-3p probe | GCAGGGCTAAACTACTACAACA |
| miRB probe | GCCGATAAGAAGGAGCCGTAA |
| novel-miR0128-5p-probe | CAGCGGTGGGCTGAGGGTAGACG |
| cre-miR910-probe | GCGGTCGAGCCCGACGCTGCT |

**Supplementary Table S5.** Determination of the fatty acid content

| The name of fatty acid | CC-5325（%） | *dcl1* （%） | *dcl3* （%） |
| --- | --- | --- | --- |
| Hexadecanoic acid, methyl ester | 12.836 | 12.681 | 14.165 |
| Methyl hexadec-9-enoate | 3.448 | 3.735 | 2.897 |
| 7,10-Hexadecadienoic acid, methyl ester | 0.900 | 0.870 | 0.775 |
| 7,10,13-Hexadecatrienoic acid, methyl ester | 1.881 | 2.092 | 1.634 |
| Methyl 4,7,10,13-hexadecatetraenoate | 16.069 | 15.658 | 15.012 |
| 9-Octadecenoic acid, methyl ester | 2.720 | 2.799 | 2.615 |
| 11-Octadecenoic acid, methyl ester | 3.644 | 3.717 | 3.792 |
| 9,12-Octadecadienoic acid (Z,Z)-, methyl ester | 6.687 | 6.669 | 6.215 |
| Methyl 5,9,12-octadecatrienoate | 5.662 | 5.716 | 5.148 |
| 9,12,15-Octadecatrienoic acid, methyl ester, (Z,Z,Z)- | 22.781 | 23.601 | 23.256 |
